# Optimizing Antifungal Treatment Strategies to Prevent Invasive Pulmonary Aspergillosis Infection-Related Deaths in Intensive Care Unit Patients: The Need for Standardization of Research Definitions

**DOI:** 10.1007/s11046-024-00879-6

**Published:** 2024-07-27

**Authors:** Matteo Bassetti, Antonio Vena, Martina Bavastro, Daniele Roberto Giacobbe

**Affiliations:** 1 Infectious Diseases Unit, IRCCS Ospedale Policlinico San Martino, Genoa, Italy; 2 Department of Health Sciences (DISSAL), University of Genoa, Via A. Pastore 1, 16132 Genoa, Italy

**Keywords:** Aspergillus, Invasive fungal diseases, Definitions, Invasive aspergillosis, ICU, IPA

## Abstract

The clinical spectrum of invasive pulmonary aspergillosis (IPA) has expanded in recent decades. A large group of patients admitted to intensive care units (ICU) is indeed susceptible to the development of IPA. Although timely diagnosis and antifungal therapy of IPA in this expanding population is crucial to prevent IPA-related deaths, the magnitude of the favorable prognostic impact of antifungal therapy is difficult to measure precisely. In our opinion, the development of standardized research definitions could have favorable implications for further improving our ability both to measure the favorable effect of antifungal treatment and to prevent IPA-related death in ICU patients.

## Introduction

Invasive pulmonary aspergillosis (IPA) is a major cause of morbidity and mortality in severely immunocompromised patients, such as patients with hematologic cancer and chemotherapy-induced neutropenia or transplant recipients [1–3]. Nonetheless, the clinical spectrum of IPA has expanded over the last decades with the emergence of new categories of patients at risk. A broad group of patients who are admitted to intensive care unit (ICU) includes nonneutropenic hosts outside classical populations at risk that are indeed susceptible to the development of IPA [4–8]. Development of IPA in nonneutropenic ICU patients has been shown to unfavorably impact their survival [4, 9, 10]. Therefore, prompt recognition and antifungal treatment of IPA in this expanding population could be crucial to prevent IPA-related deaths. However, how to use antifungals at best to curtail IPA mortality still remain largely elusive in this population, for reasons intimately connected to IPA definition.

## A Matter of Probability

Diagnostic certainty (i.e., proven diagnosis of IPA) is rarely achievable in ICU patients, since collecting pulmonary samples through biopsy is often unfeasible owing to coagulopathy, hemodynamic instability, or mechanical ventilation. Therefore, clinicians have become used to assess patients in terms of “possible”, “probable”, “putative”, or “presumptive” IPA, according to different definitions [3, 11–13]. The common point across these definitions is that IPA diagnosis, when not proven, is a matter of probability. Taking an historical perspective, we could view probability theory according to Laplace, as “*nothing but common sense reduced to calculation*” [14]. Keeping this in mind, one might reasonably hypothesize that, if we could precisely calculate the probability of IPA in a given ICU patient, we could then solidly balance, according to common sense based on the evaluation of the clinical picture and prognostic considerations, whether or not to start antifungal treatment in order to improve prognosis of true cases while at the same time avoiding widespread use of antifungals when not eventually necessary, in line with antifungal stewardship principles. However, this is all but an easy task. In this regard, almost 30 years ago Crawford and colleagues stated that “*many investigators believe that the detection of Aspergillus in the respiratory tract of a patient with significant risk factors for infection and with the appropriate clinical presentation (that is, pulmonary infiltrate) should be presumed to signify active infection, not colonization*” [15]. While this still holds true today to some extent, “presuming” an active infection involves a large spectrum of different probabilities of IPA, that often cannot be easily measured in nonneutropenic ICU patients due to broad risk factors, nonspecific clinical presentation, variable diagnostic accuracy of respiratory cultures, molecular methods, and fungal antigens, and nonspecific radiological findings (pulmonary infiltrates are shared with bacterial infections, that remain more common in many cases). For these reasons, and for the frequent lack of a large number of samples to be used as diagnostic reference standard (IPA vs. no IPA based on histology) in research studies, probability of IPA has been usually and necessarily categorized not with “risk scores” based on “points” (e.g., actually measured on regression coefficients) as frequently done for bacterial infections, but with broader, and inherently more approximated, categories of “possible”, “probable”, “putative”, or “presumptive”, mostly based on experts consensus. Consequently, defining “how much” probable remains a crucial but slipping task for IPA in nonneutropenic ICU patients, even when we label it as “probable”, “putative”, or “presumptive”. Intuitively, this influences our overall ability to improve prevention of IPA-related deaths in the ICU, by conferring some inherent and still unavoidable imprecision to our decisions about both who to treat and who to include in research studies (which also are conceptually different decisions, see below).

## The Role of Standardized Definitions in Measuring the Prognostic Impact of Antifungal Therapy

**Fig. 1:**
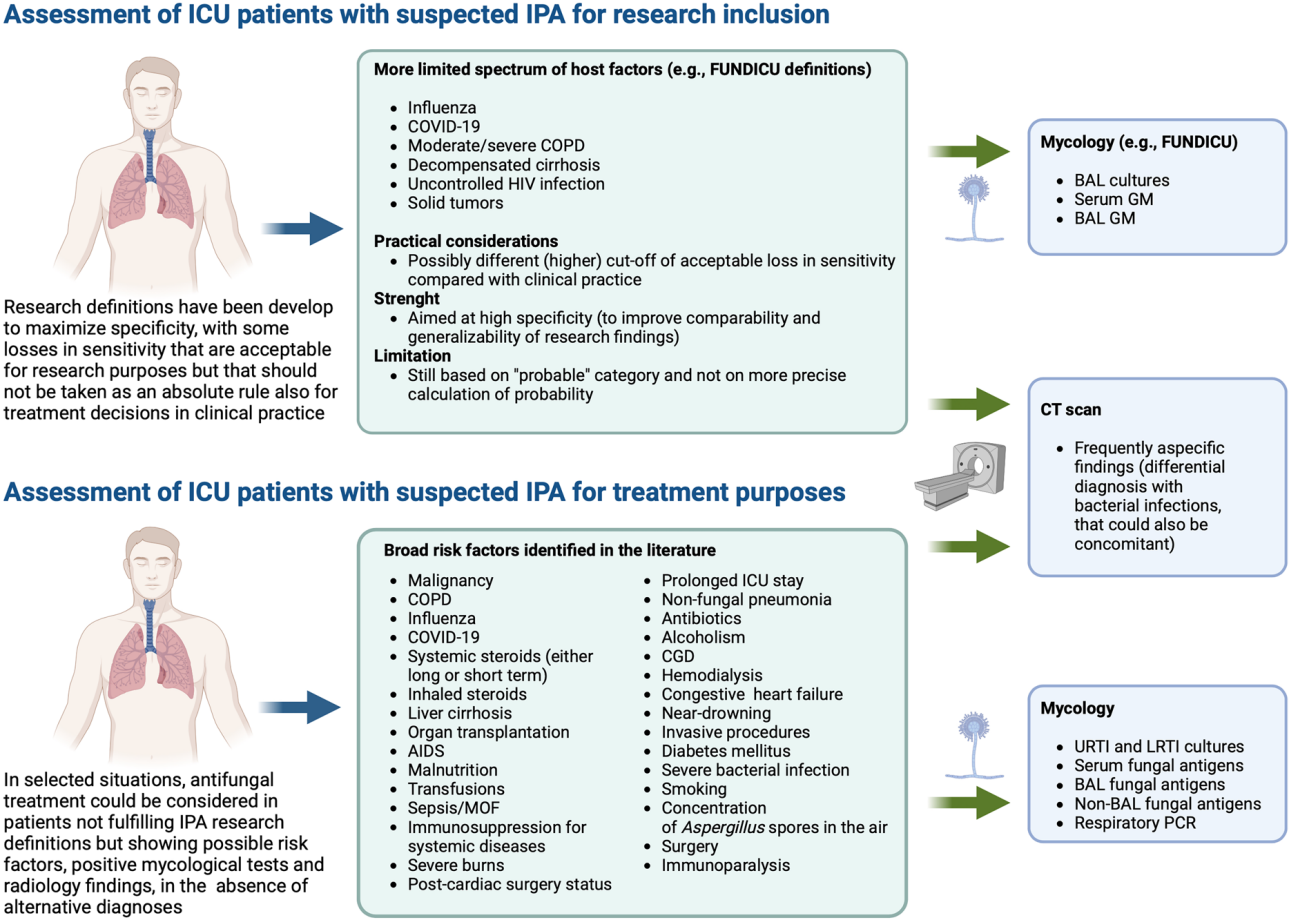
Conceptual approaches to ICU patients with suspected IPA. *AIDS* acquired immunodeficiency syndrome, *BAL* bronchoalveolar lavage, *CDG* chronic granulomatous disease, *COVID-19* coronavirus disease 2019, *COPD* chronic obstructive pulmonary disease, *CT* computerized tomography, *FUNDICU* invasive fungal diseases in adult patients in intensive care units, *GM* galactomannan, *HIV* human immunodeficiency virus, *ICU* intensive care unit, *IPA* invasive pulmonary aspergillosis, *LRTI* lower respiratory tract infection, *MOF* multiorgan failure, *PCR* polymerase chain reaction, *URTI* upper respiratory tract infection. Figure created with BioRender.com

Many definitions have been proposed or employed in research studies to classify IPA in nonneutropenic ICU patients [12, 16–26]. While certainly useful and able to prompt advancements in the field over the years, it is of note that they usually remain limited to specific categories of patients, and that they are not based on broad consensus. Although with some remaining limitations (e.g., reliance on the broad “probable” concept), the recently released FUNDICU research definitions in nonneutropenic ICU patients, jointly developed by various international scientific societies, may represent an important step forward towards providing standardization of IPA definition for research studies in nonneutropenic ICU patients, to eventually improve comparability and generalizability of research findings (including those on the impact of antifungal treatment) [27]. In this regard, a fundamental conceptual distinction should nonetheless be made between definitions developed for research purposes and decisions about whether to start or not antifungals in clinical practice, that is, research definitions are usually developed to maximize specificity, in order to increase the probability of including patients who truly have IPA in research studies, thereby reducing selection biases and consequent confounding. However, the magnitude of the losses in sensitivity that can be accepted for research purposes might conversely not always be acceptable for treatment decisions in clinical practice (see Fig. 1). For example, if a patient with suspected IPA not fulfilling research definitions (e.g., ICU host factors present, presence of positive fungal antigens but not from serum or deep respiratory samples) is clinically unstable and there are no clear alternative diagnoses, clinicians might still consider antifungal treatment despite the patient is not fulfilling (research) definitions. It should nonetheless be noted that treatment decisions in clinical practice are far less straightforward in presence of some potential predisposing factors reported in the literature (e.g., congestive heart failure, alcoholism), but for which any true association with a relevant increase in the risk of IPA is unclear (e.g., spurious associations, lack of recognition of colinearity with other true predisposing factors in statistical models, inefficient proxies for other unexplored/undetected predisposing factors, true weak association conferring a very slight increase in risk). In such cases, decisions to treat may not be supported without further advancements in precise risk definition/calculation. For other factors, the association with an increased risk of IPA could be perceived as more immediate from a causal perspective (e.g., treatment with systemic steroids). However, in similar situations uncertainty could rely on a still imprecise definition/calculation of the magnitude of the risk based on connected, relevant factors (e.g., type and duration of steroid treatment).

Keeping in mind the above premise on the conceptual distinction between research purposes and the clinical approach to treatment, it is nonetheless reasonable to suppose that standardization of IPA definition in research studies could help providing more solid comparative evidence about the positive impact of antifungal therapy in ICU patients with IPA, and also about when to start antifungal treatment to improve patients’ outcomes, information that is difficult to stem crystal clear when research studies are hardly comparable (and consequently unable to contribute forging reliable summary effects in meta-analyses). Of note, such a favorable effect would be in line with preliminary findings, with all the due limitations of the small sample, suggesting an improved survival in influenza-associated pulmonary aspergillosis (IAPA) patients who received early antifungal therapy (median 2 days after diagnosis of influenza in survivors vs. 9 days in non-survivors) [28, 29].

## Conclusion

While an unfavorable prognostic impact of IPA in nonneutropenic ICU patients has been recognized [4, 9, 10], measuring, from observational studies, the favorable impact of antifungal therapy in ICU patients with IPA has turned to be less straightforward. This does not mean that a positive effect is not present, but that its eventual magnitude is more difficult to measure, for the various reasons summarized in the previous sections. In our opinion, the lack of standardized research definitions has important implications for our ability to identify how to further optimize antifungal treatment to prevent IPA-related death in ICU patients. For example, among diagnostic and therapeutic algorithms proposed for managing coronavirus-associated pulmonary aspergillosis (CAPA) in the ICU, some differences exist about when to start antifungal therapy (e.g., empirically, after positivity of bronchoscopy or non-bronchoscopy antifungal antigens, only after positivity of bronchoscopy antifungal antigens, none of the above in the lack of new symptoms and/or presence of alternative diagnoses) that we think could reflect the current lack of solid comparative evidence [11, 30–32]. Against this backdrop, improving comparability of research studies through the development and use of standardized definitions could help improving our ability to precisely measure the impact of antifungal therapy in the heterogenous populations of ICU patients at risk, with the FUNDICU project being a first step in this direction [27]. Certainly, further improvements in definitions will likely be required in the forthcoming future, for evaluating and including any possible new evidence on the use of diagnostic technologies and artificial intelligence/machine learning-base prediction rules, should they prove able to retain (or to further increase) specificity for IPA detection with more acceptable (or even none) losses in sensitivity, eventually aiming to bring research and clinical purposes closer together (i.e., one definition for both purposes). In the meantime, clinicians and researchers approaching IPA in nonneutropenic ICU patients should continue to carefully weigh the conceptually different purposes of definitions primarily developed for research vs. algorithms aimed at supporting treatment decisions in clinical practice.

## Data Availability

No new data was generated during the preparation of the article.
